# Prognostic significance of programmed death-ligand 1 expression in betel nut chewing patients with oral squamous cell carcinoma

**DOI:** 10.1186/s12903-026-07845-2

**Published:** 2026-02-09

**Authors:** Hui-Zhu Yang, Ling-Yu Kung, Yu-Chun Lin, Cheng-Yu Yang, Gu-Jiun Lin, Yuan-Wu Chen, Ren-Hua Ye, Wei-Chin Chang

**Affiliations:** 1 School of Dentistry and Graduate Institute of Dental Science, National Defense Medical University, No.161, Sec. 6, Minquan E. Rd., Neihu Dist., Taipei, 11490 Taiwan, ROC; 2 Department of Oral and Maxillofacial Surgery, Tri-Service General Hospital, Taipei, Taiwan, ROC; 3 Department of Family Dentistry and Oral Diagnosis, Tri-Service General Hospital, Taipei, Taiwan, ROC; 4 Department of Pathology, Tri-Service General Hospital, Taipei, Taiwan, ROC; 5 School of Medicine, National Defense Medical University, Taipei, Taiwan, ROC; 6 Graduate Institute of Medical Sciences, College of Medicine, National Defense Medical University, Taipei, Taiwan; 7 Institute of Biology and Anatomy, College of Medicine, National Defense Medical University, Taipei, Taiwan; 8 Department of Medicine/Hematology and Oncology Division, Tri-Service General Hospital, Taipei, Taiwan, ROC

**Keywords:** Oral squamous cell carcinoma, Programmed death-ligand 1, Immunohistochemistry, Prognosis, Betel nut, Immune checkpoint, Combined positive score

## Abstract

**Objective:**

This study aimed to evaluate the prognostic significance of programmed death-ligand 1 (PD-L1) expression in Taiwanese patients with oral squamous cell carcinoma (OSCC) who underwent surgical treatment and had a history of betel-nut chewing.

**Materials and methods:**

We retrospectively analyzed the medical records of patients with OSCC diagnosed and treated at a single tertiary hospital between 2017 and 2023. Immunohistochemical evaluation of formalin-fixed, paraffin-embedded tumor specimens was performed using the validated anti–PD-L1 antibody clone 22C3. PD-L1 expression was evaluated using the Combined Positive Score (CPS). CPS ≥ 1 and ≥ 20 were defined as PD-L1 positivity and overexpression, respectively. An additional score was obtained by subtracting the tumor proportion score (TPS) from the combined positive score (CPS) to clarify the effect of the immune cells. The associations between PD-L1 expression, overall survival (OS), and disease-free survival (DFS) were assessed using Kaplan–Meier and Cox regression analyses.

**Results:**

Of the 118 OSCCs, 56.8% and 31.4% exhibited PD-L1 positivity and overexpression, respectively. PD-L1 overexpression and CPS-TPS ≥ 10 were significantly associated with improved OS (*p* = 0.023 and *p* = 0.035, respectively). Multivariate analysis confirmed that PD-L1 overexpression and cancer stage were independent prognostic factors for OS (*p* = 0.031 and *p* = 0.000, respectively). However, PD-L1 overexpression was not significantly correlated with tumor stage, anatomical site, or adverse pathological features.

**Conclusion:**

High PD-L1 expression is a favorable prognostic biomarker in patients with surgically treated OSCC, particularly in populations with prevalent betel nut use. These findings suggest that PD-L1 expression can guide immunotherapy decisions and risk stratification in OSCC management.

**Clinical relevance:**

Patients with OSCC exhibiting high PD-L1 expression have a better survival rate after surgery. PD-L1 testing may help in risk stratification and guiding immunotherapy decisions.

## Introduction

Oral squamous cell carcinoma (OSCC), one of the most prevalent and aggressive malignancies in the head and neck region, results in high morbidity and mortality worldwide [1, 2]. Despite advancements in surgical techniques and adjuvant therapies, the prognosis of OSCC remains poor with a 5-year survival rate of approximately 60%, particularly in patients with advanced-stage disease or those with other pathologically adverse factors [3–5]. Thus, identifying reliable prognostic factors for risk stratification and informing therapeutic decision-making is necessary.

Programmed death-ligand 1 (PD-L1), also known as CD274 or B7-H1, is a ligand of programmed death-protein 1 (PD-1) [6]. It is a transmembrane protein commonly expressed on the surface of tumor epithelial and immune cells [6, 7] and suppresses T-cell-mediated immune responses by binding to PD-1, allowing tumor cells to evade immune surveillance [3]. Elevated PD-L1 expression is associated with poor outcomes in several malignancies, including non-small cell lung cancer, advanced-stage melanoma, and gastrointestinal tumors [3, 8, 9]. However, its prognostic value in OSCC remains an area of active investigation, with several studies yielding conflicting results. Evaluation of PD-L1 expression may provide insights into the potential utility of immune checkpoint inhibitors (ICIs) as adjuvant or neoadjuvant strategies in surgically managed OSCC.

Importantly, betel nut chewing—highly prevalent in South and Southeast Asia, particularly Taiwan—has been identified as a major etiologic factor for OSCC and is associated with chronic mucosal inflammation, epithelial dysplasia, and a distinct carcinogenic pathway [10, 11]. These carcinogenic mechanisms involve persistent immune dysregulation and inflammatory cell infiltration, conditions that may influence PD-L1 expression within the tumor microenvironment. Despite this, limited studies have examined PD-L1 expression specifically in betel nut-associated OSCC, and it remains unclear whether these patients exhibit different immunologic profiles or prognostic implications compared with non–betel nut chewers. Therefore, focusing on this unique high-risk population may help clarify whether betel nut–related oncogenesis interacts with PD-L1–mediated immune escape, potentially informing tailored immunotherapeutic strategies [12].

Several scoring systems have been developed for quantifying PD-L1 expression in tumor specimens, including Tumor Proportion Score (TPS), Combined Positive Score (CPS), and Tumor Cell scoring [6, 13, 14]. TPS measures the percentage of PD-L1–positive tumor cells relative to all viable tumor cells, whereas CPS indicates the sum of PD-L1–positive tumor cells and immune cells (e.g., lymphocytes and macrophages). Conversely, the Tumor Cell score specifically evaluates PD-L1 expression in the tumor cells. These scoring systems are critical for predicting responses to ICIs and guiding cancer immunotherapy strategies [6, 15]. The choice of scoring method is often influenced by the monoclonal antibody clone used for staining, because different clones (such as 22C3 or 28 − 8) may result in distinct staining intensities and cellular localization patterns [6, 16].

This study aimed to elucidate the prognostic significance of PD-L1 expression in patients who underwent surgery for OSCC. Given the unique pathophysiology of betel nut–related carcinogenesis and its potential interaction with tumor immune evasion pathways, we specifically investigated a cohort of patients with betel nut chewing habits. By synthesizing the current evidence, we sought to clarify its role in predicting oncological outcomes and explore its implications for personalized treatment strategies.

## Patients and methods

### Selection criteria

This retrospective observational study included 118 patients with OSCC diagnosed and managed at the Tri-Service General Hospital between January 2017 and December 2023 and followed up until death or December 2024. Because betel nut chewing is a major and well-established etiological risk factor for OSCC in Taiwan, and more than 95% of our OSCC patients report habitual chewing, the study intentionally restricted inclusion to patients with a documented history of betel nut consumption (defined as chewing ≥ 1 quid per day for at least 1 year). As a result, all 118 patients (100%) included in this study were betel nut chewers and underwent curative-intent surgery consisting of wide tumor excision and neck dissection (either selective or modified radical, based on tumor stage and nodal status). Patients without a betel nut chewing habit were excluded. Additionally, patients with advanced-stage disease in whom surgery was deemed infeasible, as well as those with poor general condition, distant metastasis at diagnosis, or incomplete clinicopathological data, were excluded from the study.

### Sample preparation

Formalin-fixed, paraffin-embedded oral cancer tissue specimens were sectioned at a thickness of 4 μm using a microtome and mounted on positively charged glass slides to enhance tissue adhesion. The slides were baked at 60 °C for at least 1 hour before undergoing deparaffinization in xylene and rehydration through graded ethanol. Heat-induced antigen retrieval was performed using citrate or ethylenediaminetetraacetic acid buffer to unmask PD-L1 epitopes. Immunohistochemical staining was carried out using the PD-L1 IHC 22C3 pharmDx assay (DAKO/Agilent, Carpinteria, CA, USA) on the Dako Autostainer Link 48 platform; the primary antibody provided in the assay is in a ready-to-use format, and therefore no additional dilution was required. After staining, slides were counterstained with hematoxylin, dehydrated, and coverslipped. All stained sections were independently reviewed under a light microscope by two board-certified pathologists, and any interobserver discrepancy greater than 10% was resolved through joint evaluation using a multi-headed microscope until consensus was achieved.

### IHC evaluation

**Fig. 1:**
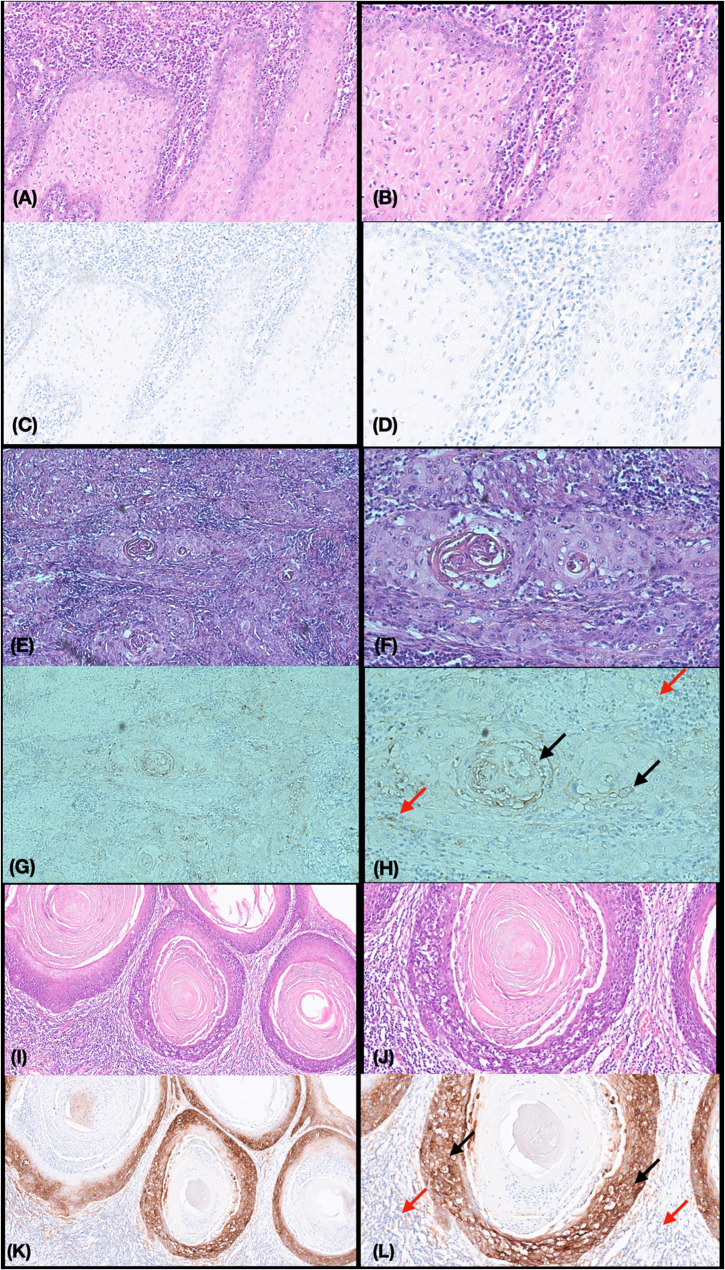
PD-L1 negative expression and overexpression. **A**–**D** Negative PD-L1 expression, showing TPS < 1% and CPS < 1. Representative HE and PD-L1 IHC staining images at ×10 (left) and ×20 (right) magnification. **E**–**H** Low-positive PD-L1 expression, with TPS = 10% and CPS = 15. At ×10 (left) and ×20 (right) magnification. In panel (H), PD-L1–positive tumor cells showing membranous staining are indicated by the black arrow (included in TPS). Peritumoral immune cells (lymphocytes and macrophages) demonstrating PD-L1 positivity are indicated by the red arrow (included in CPS but excluded from TPS). **I**–**L** High PD-L1 overexpression, with TPS = 60% and CPS = 65. Representative ×10 (left) and ×20 (right) magnification. In panel (L), intense membranous PD-L1 staining of tumor cells is marked by the black arrow. PD-L1–positive immune cells in the surrounding stroma are indicated by the red arrow, contributing to CPS assessment. TPS = Tumor Proportion Score; CPS = Combined Positive Score; HE = Hematoxylin and eosin

HPV status was determined using p16 immunohistochemistry. Strong and diffuse nuclear and cytoplasmic staining in ≥ 70% of tumor cells was considered p16-positive, consistent with established criteria for HPV-associated head and neck cancers. For PD-L1 evaluation, immunohistochemical staining was assessed at 200× magnification. The Tumor Proportion Score (TPS) was defined as the percentage of viable tumor cells exhibiting membranous PD-L1 staining at any intensity relative to the total number of viable tumor cells. The Combined Positive Score (CPS) was calculated by dividing the number of PD-L1–positive tumor cells, lymphocytes, and macrophages by the total number of viable tumor cells and multiplying by 100. Necrotic regions and staining artifacts were excluded. CPS values were categorized as < 1 (negative), ≥ 1 (positive), and ≥ 20 (overexpression), which served as the primary cutoff for prognostic grouping (Fig. 1).

An additional score was obtained by subtracting TPS from CPS, and the resulting value represents the relative contribution of immune-cell–associated PD-L1 expression within the tumor microenvironment. This parameter was used to evaluate whether PD-L1 expression was predominantly tumor-cell–driven or influenced substantially by immune-cell PD-L1 positivity, and to explore whether this imbalance was associated with differences in patient prognosis.

### Treatment and follow-up

All patients underwent therapeutic surgery, including wide tumor excision and appropriate neck dissection. Selective or modified radical neck dissection was performed based on clinical staging and intraoperative findings. Postoperative adjuvant therapy was administered according to institutional practice and in alignment with NCCN guidelines for head and neck cancers. Patients with high-risk pathological features received adjuvant radiotherapy or concurrent chemoradiotherapy, while those with intermediate-risk factors were treated with adjuvant chemotherapy alone when clinically indicated.

During the study period, immune checkpoint inhibitors (e.g., anti–PD-1/PD-L1 therapy) were not administered as routine adjuvant treatment following curative surgery. None of the included patients received immunotherapy before disease recurrence. Patients who subsequently received systemic immunotherapy for unresectable or metastatic recurrence were excluded from survival analysis to avoid confounding effects.

Patients were followed at 1–3-month intervals during the first two postoperative years and at 3–6-month intervals thereafter, or until death or the end of follow-up in December 2024.

### Survival

Patient survival data were obtained from the clinical records. The follow-up period was from the date of diagnosis to death or until December 2024. Overall survival (OS) was defined as the time from the date of diagnosis to death due to any cause. Disease-free survival (DFS) was defined as the time from the date of curative treatment to the first instance of disease recurrence (local, regional, or distant metastasis) or death from any cause.

### Statistical analysis

All statistical analyses were performed using the SPSS (version 20.0; IBM Corp., Armonk, NY, USA). The chi-square test was used to compare categorical variables, including age, sex, site, adverse factors for OSCC, alcohol consumption, cigarette smoking, and PD-L1 marker expression. Categorical variables were analyzed using Pearson’s chi-square test. Fisher’s exact test was applied only when the expected cell count was < 5. All statistical tests were two-tailed, and *p* < 0.05 was considered statistically significant. Univariate Kaplan–Meier and multivariate Cox regression analyses were performed to determine the correlation of PD-L1 overexpression with OS, and disease-free survival after treatment. Statistical significance was set at *p* < 0.05.

## Results

### Clinicopathological characteristics of the patients

This retrospective study included 118 patients, and the detailed clinicopathological characteristics of the study cohort are presented in Table 1. The cohort consisted predominantly of males (105 [89%]) and 13 females (11%). The median age at diagnosis was 57.9 years (range, 34–94 years), and the mean follow-up duration was 23.64 months (range, 0–77 months). According to the American Joint Committee on Cancer, 8th edition, most patients were diagnosed with advanced stage, like stage III or IV (82/118, 69.4%), followed by stage I or II (36/118, 30.5%). The most common primary tumor subsites were the tongue (40/118, 33.9%), followed by buccal mucosa (37/118, 31.4%) and gingiva (26/118, 22.0%).

**Table 1:** Patient demographics

Characteristics		No. of patients	Percentage (%)
Sex	Male	105	89.0
	Female	13	11.0
Mean age, years	57.9 (range, 34–94)		
HPV related status	p16 IHC (-)	106	89.8
	p16 IHC (+)	12	10.2
Tobacco exposure	No	27	22.9
	Yes	91	77.1
Alcohol exposure	No	47	39.8
	Yes	71	60.2
Betel nut exposure	No	0	00.0
	Yes	118	100.0
Margin status	≥5 mm	48	40.7
	<5 mm	65	55.0
Neck dissection	SND	55	46.6
	mRND	63	53.4
DOI	≤5 mm	36	30.5
	>5 mm	82	69.5
PNI	Negative	96	81.4
	Positive	22	18.6
LVSI	Negative	110	93.2
	Positive	8	6.8
ENE	Negative	94	79.7
	Positive	24	20.3
Overall TNM stage	I+II	36	30.5
	III+IV	82	69.4
T classification	1+2	50	42.4
	3+4	67	56.8
N classification	Nx	2	1.7
	N0	61	51.7
	N+	48	40.8
Treatment	Surgery only	28	23.7
	Surgery + RT	1	0.8
	Surgery + CT	17	14.4
	Surgery + CCRT	72	61.0
Anatomical site	Gingiva	26	22.0
	Tongue	40	33.9
	Buccal mucosa	37	31.4
	Other	15	12.6
Follow-up duration (months)	Mean: 23.64 ± 15.98		
	Median: 20		
	Range: 0–77		

*HPV* human papillomavirus, *SND* selective neck dissection, *mRND* modified radical neck dissection, *DOI* depth of invasion, *PNI*, perineural invasion; LVSI, lymphovascular space invasion; ENE, extranodal extension; TNM, tumor, node, metastasis; CCRT, concurrent chemoradiotherapy; CT, chemotherapy; RT, radiotherapy

### PD-L1 expression in OSCC was stained using 22c3

IHC staining for PD-L1 was observed in the membranes, tumor cells, lymphocytes, and macrophages. Representative images of IHC staining for PD-L1 are shown in Fig. 1. PD-L1 positivity and overexpression were observed in 56.8% (67/118) and 31.4% (37/118) of tumors, respectively. PD-L1 overexpression was observed in 55.2% (37/67) of all cases with PD-L1 positivity.

### Comparative analysis of PD-L1 expression and association with clinicopathological characteristics in OSCC

The clinicopathological characteristics of patients with OSCC, grouped according to PD-L1 expression level, are shown in Table 2. PD-L1 overexpression was not significantly associated with age, sex, anatomical site, adverse pathological factors, disease stage, cigarette smoking, or alcohol consumption. Categorical variables were analyzed using Pearson’s chi-square test or Fisher’s exact test where appropriate, based on expected cell counts. All statistical analyses were two-tailed, and *p* < 0.05 was considered statistically significant. In this study, all categorical comparisons yielded p-values greater than 0.05.

**Table 2:** Characteristics of patients of subgroups stratified according to the CPS

		CPS score		
		<20	≥20	
Variable	Total	No. of patients		*p* value
Age, years				0.300
< 65	90	64	26	
≥ 65	28	17	11	
Sex				0.495
Male	105	71	34	
Female	13	10	3	
Anatomical site				0.119
Gingiva	26	13	13	
Tongue	40	31	9	
Buccal mucosa	37	26	11	
Other	15	11	4	
DOI				0.110
≤5 mm	36	21	15	
> 5 mm	82	60	22	
Margin				0.082
Clear (≥ 5 mm)	48	28	20	
Close (< 5 mm)	65	48	17	
LVSI				0.688
Negative	110	75	35	
Positive	8	6	2	
PNI				0.647
Negative	96	65	31	
Positive	22	16	6	
Bone involving				0.376
Negative	92	65	27	
Positive	26	16	10	
ENE				0.213
Negative	94	62	32	
Positive	24	19	5	
Stage				0.379
I + II	35	22	13	
III + IV	83	59	24	
Tobacco exposure				0.489
No	27	20	7	
Yes	91	61	30	
Alcohol exposure				0.609
No	47	31	16	
Yes	71	50	21	
HPV-related status				0.617
p16 IHC (-)	106	72	34	
p16 IHC (+)	12	9	3	

*HPV* human papillomavirus, *DOI* depth of invasion, *PNI* perineural invasion, *LVSI* lymphovascular space invasion, *ENE* extranodal extension, *CPS* combined positive score

a: Pearson’s chi-square test applied (all expected cell counts ≥ 5)

b: Fisher’s exact test applied (expected count < 5). All tests were two-tailed; no comparison reached statistical significance (*p* > 0.05)

* Statistically significant difference (*p* < 0.05)

### Survival analysis

**Fig. 2:**
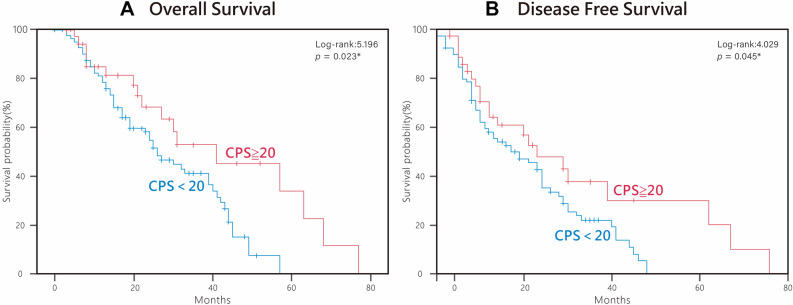
Kaplan–Meier survival curves stratified by PD-L1 expression using a CPS ≥ 20 cut-off. Kaplan–Meier analyses of patients with OSCC based on PD-L1 expression (22C3 clone), comparing tumors with CPS ≥ 20 and CPS < 20. **A** Overall survival (OS) showed a significant difference between the two groups (log-rank χ² = 5.196, *p* = 0.023). **B** Disease-free survival (DFS) similarly demonstrated improved outcomes in the CPS ≥ 20 group (log-rank χ² = 4.029, *p* = 0.045). Survival probabilities are plotted over the 5-year follow-up period

Kaplan–Meier analysis, as shown in Fig. 2, revealed that patients with PD-L1–overexpressing tumors had significantly better overall survival (OS) (*p* = 0.023). Likewise, disease-free survival (DFS) was also significantly improved in the PD-L1–overexpression group (*p* = 0.045). Univariate analysis showed that disease stage and PD-L1 overexpression were significantly correlated with OS (*p* = 0.001 and *p* = 0.023, respectively). Multivariate analysis (Cox regression model) of these factors showed that disease stage and PD-L1 overexpression were significantly correlated with OS (*p* = 0.001 and *p* = 0.010, respectively; Table 3). Furthermore, CPS-TPS ≥ 10 was significantly associated with improved OS (*p* = 0.035).

**Table 3:** Univariate and multivariate analyses of prognostic factors for 5-year OS in the entire sample

Variables	5-year survival (%)	OS			
		Univariate analysis *p* value	Multivariate analysis *p* value	HR	95% CI
Age, years		0.310	0.920		
< 65	11.9			Reference	
≥ 65	0.0			1.035	0.527–2.031
Sex		0.936	0.481		
Male	11.8			1.341	0.593–3.031
Female	0.0			Reference	
CPS score		0.023*	0.031*		
<20	0.0			2.002	1.064–3.769
≧20	34.0			Reference	
Stage		0.000*	0.000*		
I + II	28.3			Reference	
III + IV	4.7			3.167	1.667–6.015
Anatomical site		0.895	0.769		
Buccal mucosa	12.6			Reference	
Tongue	26.1		0.288	0.735	0.417–1.296
Gingiva	0.0		0.737	0.884	0.431–1.815
Others	0.0		0.694	0.862	0.412–1.804

*OS* overall survival, *HR* hazard ratio, *CI* confidence interval

* Statistically significant difference (*p* <0.05)

## Discussion

This study focused on a clinically and culturally distinct patient population. All participants were Taiwanese individuals with a history of betel nut chewing, which is a well-established risk factor for OSCC in Southeast Asia [4]. The inclusion of this relatively homogeneous cohort provides a unique opportunity to evaluate the prognostic significance of PD-L1 overexpression in high-risk groups with common environmental and behavioral exposures [17]. Considering the strong etiological association between betel nut use and oral carcinogenesis, our findings may reflect a tumor immune microenvironment specific to this subgroup, offering perspectives that differ from those of other studies based on populations without this exposure. Apart from betel nut chewing, cigarette smoking and alcohol consumption are common risk factors for OSCC [18, 19]. Some studies have reported lower PD-L1 expression in smokers due to reduced interferon-γ signaling, which is a potent PD-L1 inducer [2]. However, our results showed no significant association between cigarette smoking and OS.

Betel nut chewing has been shown to induce chronic mucosal irritation and persistent oxidative stress, leading to activation of NF-κB and STAT3 signaling pathways and consequently promoting chronic inflammation and dysregulation of local immunity within the oral cavity [20]. This inflammatory microenvironment may enhance interferon-γ secretion by activated T cells and macrophages, a well-recognized inducer of PD-L1 expression [13], which could partly explain the relatively high proportion of PD-L1 overexpression observed in our cohort (31.4%). Additionally, arecoline—the major alkaloid component of betel nut—has been reported to promote macrophage polarization toward an M2 phenotype [21], which is associated with immune cell–related PD-L1 expression. These observations support our findings that a larger TPS–CPS difference (≥ 10) correlated with improved survival, suggesting that in patients with chronic betel nut exposure, PD-L1 expression may reflect an immune-responsive rather than purely immune-evasive tumor microenvironment.

Generally, the prognosis of oral cancer is evaluated based on the presence of adverse pathological factors including extranodal extension, perineural invasion, lymphovascular invasion, depth of invasion, positive or close surgical margins, and metastatic lymph nodes [5, 22]. These histopathological features are associated with a higher risk of recurrence and poorer OS and are critical in guiding postoperative treatment decisions and prognosis assessment [3, 5, 22]. Additionally, advanced disease stage represents a worse prognosis, and the multivariate analysis in our study showed that staging was significantly correlated with OS.

In this study, we evaluated PD-L1 expression using IHC in patients with locally advanced OSCC who underwent surgical resection. Notably, patients with PD-L1 overexpression had significantly better survival outcomes than those without. This observation contrasts with previous reports suggesting that PD-L1 overexpression correlates with poor prognosis in various malignancies, such as renal cell carcinoma, colorectal cancer, and lung cancer [17]. However, studies on patients with metastatic melanoma, non-small cell lung cancer, Merkel cell carcinoma, and laryngeal cancer have shown that PD-L1 overexpression, particularly when accompanied by a high density of tumor-infiltrating lymphocytes, is associated with prolonged survival [23, 24]. To emphasize the influence of tumor-infiltrating lymphocytes, we analyzed the differences between TPS and CPS.

Previous studies focusing specifically on OSCC and head and neck cancer cohorts have also explored the prognostic impact of PD-L1 expression. Several reports have suggested that PD-L1 positivity, particularly when accompanied by a dense infiltrate of tumor-infiltrating lymphocytes, may reflect an “immune-active” tumor microenvironment and be associated with more favorable outcomes in subsets of OSCC or HNSCC patients [3, 25]. In addition, recent analyses have emphasized the importance of evaluating PD-L1 expression not only on tumor cells but also on immune cells within the stroma, as immune-cell–dominant PD-L1 staining appears to correlate with a pre-existing antitumor immune response and improved survival in some head and neck cancer cohorts [13]. These observations are in line with our finding that high PD-L1 expression, together with a higher CPS and TPS–CPS score, may indicate a more active immune microenvironment in surgically treated OSCC.

In this study, the survival rate was better in patients with PD-L1 overexpression than in those without. Conversely, most previous studies have reported an association between high PD-L1 expression and poor prognosis. Therefore, we examined the contribution of immune-cell staining to this outcome because the difference between TPS and CPS might reflect the influence of PD-L1 expression on immune cells more clearly [26]. This approach could help illustrate whether PD-L1 positivity within the tumor microenvironment, independent of tumor-cell staining, correlates with improved clinical outcomes. Previous studies have suggested that PD-L1 expression in tumor-infiltrating immune cells may reflect a preexisting antitumor immune response, which could partly explain the observed survival benefit. These findings highlight the importance of considering tumor-intrinsic and immune-related PD-L1 expression when evaluating its prognostic relevance in OSCC [14]. In this study, CPS-TPS ≥ 10 was significantly correlated with OS.

A key distinguishing feature of this study was the more stringent definition of PD-L1 overexpression adopted for patient stratification. Although most previous studies have used relatively low thresholds, commonly CPS > 1 or 5, to define PD-L1 positivity, our analysis applied a higher cut-off value of CPS ≥ 20 [2, 3, 6]. The higher threshold was selected to identify cases with biologically and clinically meaningful PD-L1 expression, thereby potentially improving the specificity of its prognostic value. By setting stricter criteria, our study aimed to better delineate the subset of patients who may truly benefit from ICIs and identify a distinct correlation between PD-L1 overexpression and clinical outcomes [27–29]. This methodological distinction may enhance the validity of our findings and contributes to ongoing discussions regarding the optimal CPS cutoff in head and neck cancer immuno-oncology research [29, 30]. However, additional research is required to establish optimal CPS thresholds for clinical application.

Immune oncology, an evolving field that uses the body’s own immune system to fight cancer, has gained significant attention as a novel treatment approach, particularly for patients with recurrent or metastatic head and neck squamous cell carcinoma [31, 32]. ICIs targeting the PD-1/PD-L1 axis have emerged as important second-line therapies, especially for patients who do not respond to standard concurrent chemoradiotherapy [20]. Nonetheless, despite its growing clinical application, the prognostic value of PD-L1 expression in advanced head and neck squamous cell carcinoma remains controversial [33].

This study had some limitations. First, the relatively small sample size of 118 patients may have limited the statistical power and generalizability of the findings. Second, because all enrolled patients were Taiwanese habitual betel nut chewers, the results reflect a highly specific high-risk population. While this homogeneity allowed focused investigation and reduced lifestyle-related confounding, it limits generalizability to non-chewing or more heterogeneous populations. As betel nut use may alter immune responses and influence PD-L1 expression, it remains uncertain whether the observed prognostic associations are due to PD-L1 overexpression alone or to interactions with betel nut–related immunomodulation. Accordingly, further validation in external cohorts with varied exposure backgrounds is needed.

## Conclusion

Our findings suggest that PD-L1 overexpression, defined as CPS ≥ 20, may serve as a favorable prognostic marker in patients with OSCC with a habit of chewing betel nuts. This finding highlights the relevance of immune checkpoint markers for risk stratification and treatment planning. Based on the unique exposure to betel nut chewing, our results offer population-specific insights. Further prospective studies are required to validate the prognostic value of PD-L1 and explore its potential as a therapeutic guide.

## Data Availability

The datasets generated and analyzed during the current study are not publicly available due to institutional and ethical restrictions. Still, they are available from the corresponding author on reasonable request and with approval from the Tri-Service General Hospital Institutional Review Board.

## Glossary

PD-L1: Programmed death-ligand 1
OSCC: Oral squamous cell carcinoma
CPS: Combined Positive Score
TPS: Tumor Proportion Score
OS: Overall survival
DFS: Disease-free survival
ICI: Immune checkpoint inhibitor
IHC: Immunohistochemical
SND: Selective neck dissection
mRND: Modified radical neck dissection
